# Supplementary material for: Variant to function mapping at single-cell resolution through network propagation
Source: Nat Biotechnol. 2022 Jun 6;40(11):1644–53. doi: 10.1038/s41587-022-01341-y (PMC9646486; doi:10.1038/s41587-022-01341-y)
Supplement: Supplementary file 1 — Reporting Summary [file 41587_2022_1341_MOESM1_ESM.pdf]

Corresponding author(s): Vijay G. Sankaran
Last updated by author(s): Apr 27, 2022

Reporting Summary

Nature Research wishes to improve the reproducibility of the work that we publish. This form provides structure for consistency and transparency in reporting. For further information on Nature Research policies, see our Editorial Policies and the Editorial Policy Checklist.

Statistics

For all statistical analyses, confirm that the following items are present in the figure legend, table legend, main text, or Methods section.

n/a	Confirmed	
☐	☒	The exact sample size (*n*) for each experimental group/condition, given as a discrete number and unit of measurement
☒	☐	A statement on whether measurements were taken from distinct samples or whether the same sample was measured repeatedly
☐	☒	The statistical test(s) used AND whether they are one- or two-sided<br>*Only common tests should be described solely by name; describe more complex techniques in the Methods section.*
☒	☐	A description of all covariates tested
☐	☒	A description of any assumptions or corrections, such as tests of normality and adjustment for multiple comparisons
☐	☒	A full description of the statistical parameters including central tendency (e.g. means) or other basic estimates (e.g. regression coefficient) AND variation (e.g. standard deviation) or associated estimates of uncertainty (e.g. confidence intervals)
☐	☒	For null hypothesis testing, the test statistic (e.g. *F*, *t*, *r*) with confidence intervals, effect sizes, degrees of freedom and *P* value noted<br>*Give P values as exact values whenever suitable.*
☒	☐	For Bayesian analysis, information on the choice of priors and Markov chain Monte Carlo settings
☒	☐	For hierarchical and complex designs, identification of the appropriate level for tests and full reporting of outcomes
☐	☒	Estimates of effect sizes (e.g. Cohen's *d*, Pearson's *r*), indicating how they were calculated

Our web collection on statistics for biologists contains articles on many of the points above.

Software and code

Policy information about availability of computer code

Data collection	No specific software was used for data collection. This is a computational method paper. Details are provided in our manuscript.
Data analysis	chromVAR_1.14.0, ArchR_1.0.1, g-chromVAR_0.3.2, FINEMAP_v1.4, GCTA-COJO_1.92.0, SCAVENGE_1.0.1, igraph_1.2.5, Matrix_1.3-4.

For manuscripts utilizing custom algorithms or software that are central to the research but not yet described in published literature, software must be made available to editors and reviewers. We strongly encourage code deposition in a community repository (e.g. GitHub). See the Nature Research guidelines for submitting code & software for further information.

Data

Policy information about availability of data

All manuscripts must include a data availability statement. This statement should provide the following information, where applicable:

- Accession codes, unique identifiers, or web links for publicly available datasets
- A list of figures that have associated raw data
- A description of any restrictions on data availability

Blood cell trait summary statistics are available from (http://www.mhi-humangenetics.org/en/resources/).
COVID-19 severity summary statistics are available from (https://www.covid19hg.org/).
The 10X PBMC dataset was downloaded from the 10X Genomics website (https://support.10xgenomics.com/single-cell-atac/datasets/1.0.1/atac_v1_pbmc_5k)
Hematopoiesis scATAC-seq datasets were downloaded from (https://github.com/GreenleafLab/MPAL-Single-Cell-2019) and (https://github.com/GreenleafLab/10x-scATAC-2019).
The analyzed data are publicly available at (https://github.com/sankaranlab/SCAVENGEreproducibility).

Field-specific reporting

Please select the one below that is the best fit for your research. If you are not sure, read the appropriate sections before making your selection.

☒ Life sciences ☐ Behavioural & social sciences ☐ Ecological, evolutionary & environmental sciences

For a reference copy of the document with all sections, see nature.com/documents/nr-reporting-summary-flat.pdf

Life sciences study design

All studies must disclose on these points even when the disclosure is negative.

Sample size	There are 5,335 cells included in 10X PBMC dataset from one human sample, 35,038 cells in hematopoiesis dataset from 10 human samples, 63,882 cells in hematopoiesis dataset 2 from 19 human samples and 97,315 cells in COVID-19 PBMC dataset from 11 human samples. Please refer to original publications for more details.
Data exclusions	There were 773 cells in 10X PBMC dataset and 1,229 cells in hematopoiesis dataset were excluded for analyses due to low quality or unknown labels (Methods).
Replication	There was no replications of samples in the datasets we analyzed.
Randomization	There was no randomization of samples in the datasets we analyzed.
Blinding	There was no blinding in the datasets we used.

Reporting for specific materials, systems and methods

We require information from authors about some types of materials, experimental systems and methods used in many studies. Here, indicate whether each material, system or method listed is relevant to your study. If you are not sure if a list item applies to your research, read the appropriate section before selecting a response.

Materials & experimental systems

n/a	Involved in the study
☒	☐ Antibodies
☒	☐ Eukaryotic cell lines
☒	☐ Palaeontology and archaeology
☒	☐ Animals and other organisms
☒	☐ Human research participants
☒	☐ Clinical data
☒	☐ Dual use research of concern

Methods

n/a	Involved in the study
☒	☐ ChIP-seq
☒	☐ Flow cytometry
☒	☐ MRI-based neuroimaging
